# Dr. Henry Raymond Rogers

**Published:** 1901-11

**Authors:** 


### OBITUARY NOTES.

Dr. Henry Raymond Rogers, of Dunkirk, N.Y., died at his home October 19, 1901, aged 79 years. He graduated in medicine at Jefferson Medical College, Philadelphia, in 1851, and was noted for his literary culture and scientific attainments.

